# Challenges of Engaging Primary Care Providers in Specialized Telementoring Education About Sickle Cell Disease for Sickle Cell Specialists: Results from the Sickle Cell Disease Training and Mentoring Program for Primary Care Providers (STAMP) Project ECHO

**DOI:** 10.1016/j.focus.2024.100304

**Published:** 2024-11-22

**Authors:** Lisa M. Shook, Bailey House, Christina Bennett Farrell, Rosalyn Stewart, Sophie Lanzkron, Allison A. King, Taniya Varughese, J.J. Strouse, Marsha Treadwell, Julie Kanter

**Affiliations:** 1 Department of Medicine, University of Cincinnati College of Medicine, Cincinnati, Ohio; 2 Division of Hematology, Cincinnati Children's Hospital Medical Center, Cincinnati, Ohio; 3 Department of Medicine Johns Hopkins School of Medicine, Baltimore, Maryland; 4 Department of Pediatrics, Washington University School of Medicine, St Louis, Missouri; 5 Department of Pediatrics, Duke University, Durham, North Carolina; 6 Department of Pediatrics, University of California, San Francisco, California; 7 Department of Medicine, University of Alabama, Bermingham, Alabama

**Keywords:** Sickle cell disease, continuing education, telementoring, chronic disease

## Abstract

**Introduction:**

Sickle cell disease (SCD) is an inherited blood disorder affecting approximately 100,000 individuals in the U.S. A lack of knowledgeable providers, particularly for adult patients, has led to a significant number of adults without access to high-quality care. Several federal partners collaborated with the Sickle Cell Disease Treatment Demonstration Program (SCDTDP) grantees to develop and test a national Project ECHO telementoring program targeting primary care providers (PCPs).

**Methods:**

Federal partners developed an extensive recruitment and outreach strategy to engage PCPs in the Sickle Cell Disease Training and Mentoring (STAMP) virtual educational (telementoring) program using a multi-pronged recruitment strategy. The SCDTDP grantees created a tailored curriculum of didactic presentations about the management of SCD, especially for PCPs.

**Results:**

STAMP hosted 12 sessions over 6 months. Despite the multi-pronged recruitment strategy and tailored curriculum, there were very few PCPs among the 763 attendees. The majority of attendees were hematologists and other community-based advocates with a vested interest in SCD.

**Conclusions:**

Despite a federal partnership, the STAMP outreach strategy was unsuccessful in recruiting PCPs to participate in a virtual telementoring education program designed to increase access to care for adults with SCD. STAMP's attendee participation and the lack of engagement by PCPs were unable to significantly show an increase in interest or willingness by PCPs to learn to co-manage SCD care with specialists.

## Highlights

• There is a lack of primary care providers who are knowledgeable about sickle cell disease.
• STAMP ECHO was designed to educate primary care providers about sickle cell disease.
• A national recruiting strategy was implemented to recruit participants.
• STAMP ECHO telementoring had very limited uptake by primary care providers.

## INTRODUCTION

Sickle cell disease (SCD) is the most common blood disorder in the U.S., affecting over 100,000 people.^1^ SCD can result in chronic organ complications, significant morbidity, and early mortality. Previously a pediatric condition, most affected individuals in the U.S. are now surviving into adulthood and require disease-specific care to address complications and manage treatment, preventive care, and other conditions associated with aging, such as diabetes, obesity, and cancer.

There is a paucity of hematologists to care for adults with SCD. Reasons include insufficient federal funding, insufficient reimbursement by third-party payers, a lack of hospital support for non-billing providers, and the need for supportive care team members.^2^^,^^3^ There is an insufficient number of primary care providers (PCPs) available to treat adults with SCD independently or in collaboration with an SCD provider, mostly because of the discomfort in managing SCD.^2^ Additional studies have shown that even when PCPs were willing to care for affected adults, they were not knowledgeable about the guidelines and recommendations available.^4^ Furthermore, many adults with SCD face difficulty in care management because of poor coordination between primary and subspecialty care.^5^, ^6^, ^7^

To improve access to primary care, the Health Resources and Services Administration (HRSA) proposed that the Sickle Cell Treatment Demonstration Project (SCDTDP) grantees educate adult PCPs to manage SCD. Although previous studies did not reveal enthusiasm for the care of people with SCD, one specific study did highlight the interest in learning by PCPs who felt unaware of recent guidelines.^7^ Furthermore, the 5 SCTDP regions had already launched region-specific Project ECHO (Extension for Community Healthcare Outcomes) telementoring to provide evidence-based SCD education. The ECHO model was initiated before this effort because of its success with educating PCPs about comanagement in other chronic disorders.^8^ However, participants attending the regional SCD ECHOs were often hematologists and rarely PCPs. Thus, in 2019, the HRSA SCDTDP grantees collaborated with the Office of Minority Health, Office of the Assistant Secretary for Health (OASH), to create the Sickle Cell Disease Training and Mentoring Program for Primary Care Providers” (STAMP) to target PCP engagement with Project ECHO. The goals for STAMP were to identify PCPs interested in caring for patients with SCD in partnership with specialists, to increase PCPs’ awareness and willingness to prescribe hydroxyurea therapy, and to increase PCP participation in SCDTDP TeleECHOs.

## METHODS

The target audience for the STAMP ECHO program were PCPs and other clinicians directly involved in the clinical management of adults with SCD. This was a multi-pronged, very broad recruitment strategy focused on contacting clinicians across the country with specific concentration in areas of known high populations of those with SCD or those with large African American or Hispanic populations. Participant recruitment and outreach was primarily conducted by the Health and Human Services Regional Minority Health Coordinators, OASH, members of the Health and Human Services SCD Workgroup's Clinical Subgroup, and staff from the HRSA Bureau of Primary Health Care. The Office of Minority Health targeted recruitment of PCPs through HRSA-supported community health centers, federally qualified health centers (FQHCs), physician assistant and nurse practitioner graduate programs, physician residency training programs, and through multiple national and state-led professional societies. Additional recruitment support was provided by the HRSA's SCDTDP and the Newborn Screening and Follow-up Program grantees. Outreach was conducted through website promotion, emails, and direct phone calls to professional organizations, clinics, Historically Black Colleges and University staff, and individual providers.

Recruitment was conducted at 3 levels: national, regional/state, and local. (Appendix Table 1, available online). Over 30 national organizations prioritized, along with multiple state and regional organizations, prioritized the population of people with SCD estimated to live in the state or region based on estimated patient population data.^9^ National outreach was primarily conducted by emails sent from the OASH's office with follow-up contact by the Bureau of Primary Health Care. Although over 10,000 emails were sent to these national organizations, SCTDP grantees were not informed of the number of responses received by the OASH. The OASH and the Office of Minority Health promoted the program through their individual websites and jointly created a website that housed a STAMP registration link. There were multiple national webinars during which the STAMP program was advertised, including the HHS-sponsored Sickle Cell Disease Stakeholder Engagement Webinar (November 2019), the HRSA Bureau for Primary Health Care All-program's webcast (November 2019), and STAMP was highlighted in the primary health care digest. Social media was not used to any significant extent. The regional or state outreach was focused, and each regional minority health coordinator designee coordinated with their regional SCDTDP grantee to co-develop outreach strategies. Local outreach used a targeted, high-touch strategy to identify providers or organizations most likely to serve patients with SCD in high-population cities.

Regional minority health coordinators developed region-specific spreadsheets of providers for priority outreach and used a common tracking system. Regional minority health coordinators followed a systematic approach of contacting providers or organizations through direct calls, emails, in-person visits, and/or serial outreach. Emails were sent to over 15,000 individuals through these regional strategies, though the percentage of responses was not quantified. Many emails were sent to regional and local community residency programs, FQHC leads, and to Historically Black Colleges and University nursing and student leads. Each regional minority health coordinator or regional designees shared results of outreach during Office of Minority Health SCD Workgroup Chair monthly meetings. Appendix Table 2 (available online) shows the list of regional contacts contacted in the northeast SCTDP's catchment area as an example of the breadth of societies and contacts included in this effort.

SCDTDP grantees from all 5 regional networks—Sickle Cell Improvement Across the Northeast Region through Education; Embrace Education and Mentoring to Bring Access to Care for Sickle Cell Disease in the southeast region; Sickle Treatment and Outcomes Research in the Midwest; the Heartland and Southwest Sickle Cell Disease Network; and the Pacific Sickle Cell Regional Collaborative—partnered to design the STAMP curriculum based on their SCD expertise and experience with regional Project ECHOs.

The STAMP curriculum was designed for PCPs and was informed by a brief survey of 15 PCPs, with 53% of participants reporting that they felt they had vague or slight knowledge skills and competence about SCD; less than half of the participants could recognize patients eligible for hydroxyurea; 67% self-reported they had no, slight, or vague knowledge of initiating hydroxyurea, whereas 73% self-reported even less competence in titrating hydroxyurea or other disease-modifying therapies. Only 27% of participants felt competent as a provider for patients with SCD, with only 20% reporting competence managing chronic pain. STAMP ECHO included didactic topics tailored to PCPs for evidence-based co-management of SCD, including the pathophysiology of SCD; hydroxyurea for adults; imaging uncomplicated headaches in SCD; screening assessments; transfusion; new medications for SCD; pain management; self-management techniques for adults; common lab findings; contraceptives; and telemedicine.

STAMP ECHO sessions were designed following the Project ECHO framework of an hour-long virtual session using Zoom that included an evidence-based didactic presentation followed by a de-identified patient case presented by a PCP attending the session.^10^ The key feature of ECHO sessions is the presentation of real-world clinical cases (usually presented by attendees) to direct learning and education. Template case forms were developed for completion by the presenter to ensure inclusion of relevant clinical and psychosocial patient details. Case presentations were offered as a voluntary opportunity for PCPs to get real-time feedback and support from subject matter experts on medical and psychosocial evidence-based management of SCD for complex patients. Continuing medical education credits, including Maintenance of Certification Part II credits from the American Board of Pediatrics and the American Board of Internal Medicine were offered for each session to incentivize PCPs.

The program success was measured by attendance of PCPs at the STAMP ECHO sessions and not by the quantity of email responses. As the goal of the program was PCP engagement in STAMP, it was important to measure outcomes by STAMP attendance. Demographic information collected during online registration from participants included type of practice, type of provider, and if the providers had a history of treating people with SCD. The number of STAMP ECHO sessions that each participant attended was tracked, as well as if participants followed up with their regional SCDTDP programs. A satisfaction evaluation survey including a request for feedback was sent to all participants that attended at least one session.

## RESULTS

**Figure 1:**
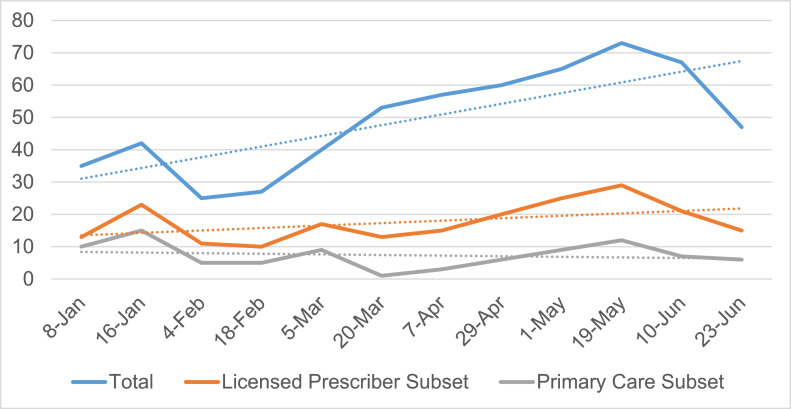
Sickle Cell Disease Training and Mentoring Program for Primary Care (STAMP) attendance over time.

STAMP ECHO launched in January 2020 with a total of 12 sessions over a 6 month period. STAMP sessions were an hour long with varying start times (11am EST – 5pm EST) to meet the needs of providers across multiple time zones and with varying clinical responsibilities. The timing was recommended by the Bureau of Primary Health Care to include options before and after clinic and during the lunch hour. Collectively, there were 537 attendees across the 12 sessions. Total attendance is shown in Table 1 and Figure 1. The majority of unique attendees were not PCPs, physicians, or advanced practice providers (APPs) but were other healthcare professionals (nurses, social workers, care coordinators) or patient advocates from several community-based organizations (81%), as shown in Figure 1 and Table 2.

**Table 1:** Total STAMP Attendees by Session

Session	Partners/presenters *n* (%)	Attendees *n* (%)	Total
January 8	17 (33)	35 (67)	52
January 16	20 (32)	42 (68)	62
February 4	14 (36)	25 (64)	39
February 18	15 (36)	27 (64)	42
March 5	12 (23)	40 (77)	52
March 20	16 (23)	53 (77)	69
April 7	14 (23)	47 (77)	61
April 29	11 (15)	60 (85)	71
May 1	13 (17)	65 (83)	78
May 19	15 (17)	73 (83)	88
June 10	20 (23)	70 (77)	90
June 23	12 (20)	47 (80)	59
Total	167 (24)	537 (76)	763

STAMP, Sickle Cell Disease Training and Mentoring Program for Primary Care.

**Table 2:** Unique Sickle Cell Disease Training and Mentoring Program for Primary Care (STAMP) Attendees by Provider Type

Type	Licensed prescribers (PCP+Specialists)	All PCPs subset	Advanced practice provider PCP subset	Physician PCP (MD,DO) subset	All other	Total
Registered	127	63	33	30	330	457
Attended	86	41	23	18	134	220
Repeated attendance	49 (59%)	23 (56%)	11 (48%)	12 (67%)	62 (46 %)	111 (50%)
Sessions stats	2 median 2.59 mean 1-9 range		1.5 median 1.65 mean 1-3 range	3 median 2.69 mean 1-5 range	2 median 2.35 mean 1-10 range	2 median 2.47 mean 1-10 range

DO, Doctor of Osteopathic Medicine; MD, Medical Doctor; PCP, Primary Care Provider.

PCPs in attendance (physicians and APPs) ranged from 1 to 15 participants at each session (mean 7.3 PCPs per session, median 6.5 PCPs per session), with overall participants attending sessions ranging from 39 to 99. PCP attendance waned over time (Figure 1). Post-ECHO survey responses showed that PCPs attending STAMP had varied previous experience with treating SCD and saw a range of the number of individuals with SCD in their practices, with an average of 32 patients per practice with SCD seen in the previous 12 months. The clinical time of each PCP is not known, as that was not included in the assessment survey. The PCP participants represented 21 states in the U.S.; Washington, D.C.; Canada; and Ghana. Other non-PCP attendees represented 33 states in the U.S. and 10 countries (Greenland, Ghana, India, Jamaica, Nigeria, Uganda, Tanzania, United Kingdom, Canada, and North Macedonia).

Evaluation data captures a sample of registrants for STAMP, as only 83 participants completed an evaluation survey. Of the 83 participants who completed a survey, only 8 were PCPs who were outpatient providers, and 75% (*n*=6) reported working in an FQHC or look-alike clinic. No providers indicated practicing in a rural zip code. Similarly, only 8 attendees stated they would plan to come to future STAMP ECHO sessions, and 8 attendees noted that they would recommend the STAMP ECHO to their colleagues. The majority felt the didactic and case presentations were adequate for their learning style, and many licensed prescribers reported attending other regional SCD ECHOs hosted by the regional SCDTDP programs.

Despite significant efforts to encourage PCP participation and engagement in STAMP ECHO, only one de-identified patient case was voluntarily presented by a STAMP attendee over 12 session opportunities. In several instances, SCDTDP leads personally emailed PCPs to request that they present a patient case to the ECHO, but the PCP was usually only interested in attending (if at all). In the absence of PCPs volunteering to present cases, the SCD expert team leading the specific didactic session developed and presented teaching cases for discussion.

## DISCUSSION

The STAMP program was specifically designed to educate and engage PCPs in the co-management of adults with SCD alongside specialists. Despite a multipronged approach by federal and regional programs reaching out to thousands of PCPs, only 220 unique participants ultimately attended. Of those, a mere 41 physicians and advanced practice providers practicing primary care attended the STAMP Project ECHO, and the majority only attended 1 session. Instead, the majority of attendees included a variety of healthcare workers (nurses, care coordinators, social workers) and community members (i.e., patient advocates and non-medical community-based organization members) but did not successfully maintain a focus on PCP participants. Thus, STAMP did not fulfill its goal of engaging and educating large numbers of PCPs about evidence-based SCD care for which it had been designed.

There were several limitations to this project. The recruitment was very broad but was not always personalized to individual providers (especially on the federal level). Instead, many of these emails and phone calls were made to program or association leads who then disseminated the information to their audiences. Thus, it is possible that the use of associations and provider groups is not optimal for advertising educational initiatives. Another limitation was the paucity of PCPs that completed the initial needs assessment survey, limiting the ability to optimally tailor the education to their needs. There were also very limited numbers of PCPs who attended the STAMP sessions that completed evaluation surveys. As a result, feedback was minimal, and it is difficult to make conclusions. STAMP may have benefited from a PCP champion within each primary care professional organization to endorse the program. Although emails clearly were exchanged between national, regional, and local leaders of these organizations and the OASH representatives, it may not have been sufficient for culture change. The effort was highly supported by both HRSA and Bureau for Primary Health Care leadership, including the design of a project, specific website by the Office of Minority Health to attempt to increase access to providers knowledgeable about SCD. Although there were limitations, this project was an important, widespread, federally supported effort to engage PCPs in continuing education about SCD on a national level.

It is also important to note that STAMP was advertised broadly in the fall and winter of 2019 and launched in January 2020 for 6 months. The second half of this pilot was conducted at the beginning of the COVID-19 pandemic. Although it is not possible to calculate the effect of the pandemic on this program, it was widely recognized that fewer people saw patients in the office during 2020, suggesting that additional time may have been available for educational programs such as STAMP. The pandemic may have been the reason the authors see a slight increase in engagement in Spring of 2020, though it was not sustained.

Whereas STAMP did recruit new individuals to the overall efforts to increase access to evidence-based SCD care, STAMP failed to substantially engage a significant number of PCPs. The primary goal of STAMP was to increase PCP engagement to improve co-management of adults with SCD; this was not successful. Within the SCTDP, there are more than 50 SCD specialists. There were no additional PCP-hematology partnerships made during this program, suggesting that the program did not increase access to care. Although there are limitations to the recruitment process, the national support from the Bureau of Primary Health Care should have resulted in more engaged PCPs based on volume and reach. Further, STAMP results did not show active engagement by PCPs to participate in continuing medical education to learn to co-manage SCD with specialists, despite personal attempts at the local level in addition to the broader recruitment efforts noted.

Importantly, Project ECHO sessions are designed for providers to present de-identified case presentations about those complex patients for whom they have medical or psychosocial management conundrums. In addition to learning, these case presentations give providers an opportunity for real-time feedback. ECHO sessions not only provide telementoring support but also promote learning in 3 unique ways: long-term co-management of complex patients between expert and provider participants; learning from fellow participants, including observing cases presented by other learners and participating in case-based discussions; and didactic lectures that provide further detail on various aspects of care for the disease.^10^ This framework is what has made Project ECHO successful in the management of hepatitis C and many other complex acute and chronic diseases. However, across 12 STAMP sessions, only one PCP presented a patient case, indicating a lower level of engagement and creating a missed opportunity for richer and more engaging discussions for all participants or to develop a community of practice.

The STAMP program was a lost opportunity to create a hub–spoke model of care or a community of practice with a partnership between SCD specialists and PCPs. There are several lessons to be learned from this effort. SCD is a complicated, complex chronic condition that requires a multidisciplinary team. There are many other diseases in which patients from rural and hard-to-reach communities are affected, and the solution has been to bring rural expertise to the patients and not to depend on the PCP to manage these complex individuals’ specialty care (e.g., hemophilia, cystic fibrosis, and cancer).^11^ In these situations, the role of the PCP has been to ensure individuals with high-risk chronic disease are assessed regularly by a specialist treating that disorder. This program's failure to engage PCPs has shown that there is a need to put the same solution in place for those with SCD to ensure funding and support to bring the patient to the nearest specialty clinic. Although there were multiple limitations in the recruitment of PCPs in the STAMP program, there was significant federal and regional support by PCP-focused organizations to encourage engagement and participation. The failure of the STAMP program to even moderately engage or interest PCPs in SCD care education highlights an ongoing failure of our healthcare system to provide care for this at-risk population. Multiple sessions were allotted for STAMP to ensure that anyone with interest could attend at least 1 program. Furthermore, the Bureau for Primary Health Care recommended the times for this program; however, despite tailoring both education and timing of the program for the PCPs, there was little to no interest.

## CONCLUSIONS

These results highlight the need for additional federal funding dedicated to SCD centers to enhance access to high-quality, evidence-based care for those individuals living with SCD, building models to extend care to more rural clinics, and providing transportation for people to receive the care. These interventions should be in collaboration with PCPs but dedicated to the SCD centers who are dedicated to this patient population. Additional methods of improving care-partnerships include embedding a PCP in the SCD center, increasing SCD-specific affiliate spokes in rural communities, and forming partnerships with community-based organizations to ensure patients can get to the care they deserve. The person living with SCD must be the focus and should receive care from an expert multidisciplinary SCD team, as with any other complex disease.

## Appendix Supplementary materials

Image, application 1

Image, application 2

## Footnotes

Supplementary material associated with this article can be found in the online version at doi:10.1016/j.focus.2024.100304.
