# Supplementary material for: Challenges of Engaging Primary Care Providers in Specialized Telementoring Education About Sickle Cell Disease for Sickle Cell Specialists: Results from the Sickle Cell Disease Training and Mentoring Program for Primary Care Providers (STAMP) Project ECHO
Source: AJPM Focus. 2024 Nov 22;4(1):100304. doi: 10.1016/j.focus.2024.100304 (PMC11758125; doi:10.1016/j.focus.2024.100304)
Supplement: Supplementary file 1 [file mmc1.docx]

Appendix Table 1: STAMP Recruitment efforts at the National, Regional, and Local levels

| National | American Academy of Family Physicians  American Academy of Physician Assistants  American Association of Nurse Practitioners  American Psychiatric Association  American Psychological Association  American Board of Emergency Medicine  American Board of Family Medicine  American Board of Internal Medicine  American College of Emergency Physicians  (Emergency Department Sickle Cell Care Coalition)  American College Health Association  (Ethnic Diversity Coalition)  American College of Obstetrics and Gynecology  American College of Physicians  American Nurses Association  American Society of Hematology  Association of Clinicians for the Underserved (ASU)’s Star Center*  Association of Nigerian Physicians in the Americas  Black Nurses Rock  Community Health Center, Inc.*  Emergency Nurses Association  HHS Center for Faith and Opportunity Initiatives  HRSA Bureau of Primary Health Care  HRSA Bureau of Health Workforce  National Association of Community Health Centers*  National Association of Community Health Workers  National Association of Hispanic Nurses  National Association of Nigerian Nurses in North America  National Association of School Nurses  National Association of Social Workers  National Athletic Trainers’ Association  National Center for Health in Public Housing*  National Hispanic Medical Association  National Nurse-Led Care Consortium*  National Medical Association  Public Health Foundation  School-based Health Alliance*  Sickle Cell Community Consortium  Sickle Cell Disease Association of America  Society of Hospital Medicine  White House Initiative on Educational Excellence for Hispanics  White House Initiative on Historically Black Colleges and Universities (HBCUs) |
| --- | --- |
| Regional/State (focus on the top 20 states with highest estimated SCD population Brousseau 2010) | State and Regional Primary Care Associations (*facilitated by BPHC via newsletter and targeted emails)*  Area Health Education Centers  Faith-based organizations  State SCD advocacy groups  State Medicaid Offices and Directors  State Managed Medicaid Health Plan Directors  State Minority Health POCs  State offices/affiliates of national organizations listed above |
| Local | Federally qualified health centers (FQHCs) located in targeted cities:   - - List of mid-sized cities^+^ with large African American population (n=42 total)   - Top 10 cities by total population and percentage of African Americans (per 2010 Census)   - Top 10 cities by total population of Hispanics (per 2010 Census)   HBCU student health clinics (focus on HBCUs with >3K total African American student enrollment [n=26]) based on the 2017 HBCU enrollment numbers  Local SCD advocacy groups serving the top 10 largest cities and 42 mid-sized cities  Advanced Practice Provider (APP) training programs in mid-sized cities (n=42 cities)  Internal Medicine/Family Medicine residency programs in mid-sized cities (n=42 cities)  Lists of SCD primary care providers shared by SCD advocacy groups |

*Organization that has National Cooperative Agreement with Bureau of Primary Health Care (BPHC)

+ Mid-Sized Cities are defined as total population 250K-500K plus African American population of at least 30K
