# Supplementary material for: Challenges of Engaging Primary Care Providers in Specialized Telementoring Education About Sickle Cell Disease for Sickle Cell Specialists: Results from the Sickle Cell Disease Training and Mentoring Program for Primary Care Providers (STAMP) Project ECHO
Source: AJPM Focus. 2024 Nov 22;4(1):100304. doi: 10.1016/j.focus.2024.100304 (PMC11758125; doi:10.1016/j.focus.2024.100304)
Supplement: Supplementary file 2 [file mmc2.docx]

Appendix Table 2: Example of regional contacts for STAMP as used by the Northeast HRSA SCTDP which includes DC, DE, MD, MA, NJ, NY, PA, RI, and VA

| Type of organization | Location | City | State |
| --- | --- | --- | --- |
| Health Professional Assn | New England Regional BNA | Roxbury | MA |
| Health Professional Assn | Western Massachusetts Black Nurses Association | Springfield | MA |
| Health Professional Assn | Black Nurses Association of Greater Washington, DC Area | Washington | DC |
| Health Professional Assn | Downtown Baltimore Black Nurses Association | Baltimore | MD |
| Health Professional Assn | Black Nurses Association of Baltimore | Baltimore | MD |
| Health Professional Assn | Greater Bowie Maryland NBNA Chapter | Chevy Chase | MD |
| Health Professional Assn | Black Nurses of Southern Maryland | Temple Hills | MD |
| Health Professional Assn | New Jersey Integrated Black Nurses Association | Lyons | NJ |
| Health Professional Assn | Northern New Jersey Black Nurses Association | Newark | NJ |
| Health Professional Assn | New York Black Nurses Association | White Plains | NY |
| Health Professional Assn | Greater New York City - Black Nurses Association | Brooklyn | NY |
| Health Professional Assn | Rochester Black Nurses Association | Rochester | NY |
| Health Professional Assn | Suffolk County Black Nurses Association | Ridge | NY |
| Health Professional Assn | Pittsburgh Black Nurses In Action | Pittsburgh | PA |
| Health Professional Assn | Southeastern Pennsylvania Area Black Nurses | Philadelphia | PA |
| Health Professional Assn | Black Nurses Association of Charlottesville, Virginia, Inc | Charlottesville | VA |
| Health Professional Assn | Central Virginia Chapter National Black Nurses Association | Richmond | VA |
| Health Professional Assn | Northern Virginia Chapter, National Black Nurses Association | Woodbridge | VA |
| Health Dept | MA Dept of Public Health/Office of Health Equity | Boston | MA |
| Health Dept | RI Dept of Public Health/Office of Health Equity | Providence | RI |
| Health Dept | NJ Dept of Health/OMMH | Trenton | NJ |
| Health Dept | NY State Dept of Health/OMHHDP | Albany | NY |
| PCP (Other/Non-FQHC) | MGH | Boston | MA |
| PCP (Other/Non-FQHC) | BMC | Boston | MA |
|  |  |  |  |
| Health Professional Assn | District of Columbia Primary Care Association | Washington | DC |
| Health Professional Assn | Massachusetts League of Community Health Centers | Boston | MA |
| Health Professional Assn | Maryland (Also see Mid-Atlantic Association of Community Health Centers (Maryland and Delaware) | Lanham | MD |
| Health Professional Assn | New Jersey Primary Care Association | Hamilton | NJ |
| Health Professional Assn | Community Health Care Association of NYS | New York | NY |
| State/Regional Org | PA Association of CHCs | Wormleysburg | PA |
| Health Professional Assn | VA Community Healthcare Association | Richmond | VA |
| Health Professional Assn | NASW DC Metro Chapter | Washington | DC |
| Health Professional Assn | NASW Massachusetts Chapter | Boston | MA |
| Health Professional Assn | NASW Maryland Chapter | Baltimore | MD |
| Health Professional Assn | NASW New Jersey Chapter | North Brunswick | NJ |
| Health Professional Assn | NASW New York State Chapter | Albany, | NY |
| Health Professional Assn | NASW Pennsylvania Chapter | Mechanicsburg | PA |
| Health Professional Assn | NASW Virginia Chapter | Glen Allen | VA |
| Health Professional Assn | Delaware Chapter ACEP |  | DE |
| Health Professional Assn | District of Columbia Chapter ACEP |  | DC |
| Health Professional Assn | Maryland Chapter ACEP |  | MD |
| Health Professional Assn | Massachusetts College of Emergency Physicians |  | MA |
| Health Professional Assn | New Jersey Chapter ACEP |  | NJ |
| Health Professional Assn | New York Chapter ACEP |  | NY |
| Health Professional Assn | Pennsylvania College of Emergency Physicians |  | PA |
| Health Professional Assn | Virginia Chapter ACEP |  | VA |
| Health Professional Assn | Maryland Academy of Family Physicians |  |  |
| IM/FM/APP | Providence Hospital Program | Washington | DC |
| IM/FM/APP | MedStar Health (Baltimore) Program | Baltimore | MD |
| IM/FM/APP | Sinai Hospital of Baltimore Program | Baltimore | MD |
| IM/FM/APP | St Agnes HealthCare Program | Baltimore | MD |
| IM/FM/APP | University of Maryland Prince George's Hospital Center Program | Cheverly | MD |
| IM/FM/APP | University of Maryland Prince George's Hospital Center Program | Cheverly | MD |
| IM/FM/APP | Capital Health Regional Medical Center Program | Trenton | NJ |
| IM/FM/APP | Seton Hall University-Hackensack Meridian School of Medicine (St Francis) Program | Trenton | NJ |
| IM/FM/APP | Carilion Clinic-Virginia Tech Carilion School of Medicine Program | Roanoke | VA |
| IM/FM/APP | Carilion Clinic-Virginia Tech Carilion School of Medicine Program | Roanoke | VA |
| IM/FM/APP | LewisGale Medical Center Program | Roanoke | VA |
| FQHC | Southern Jersey Family | Hammonton | NJ |
| FQHC | Henry J Austin Health Center Inc | Trenton | NJ |
| FQHC | Three Lower Counties Community Services Inc | Salisbury | MD |
| FQHC | Kuumba Community Health & Wellness Ctr | Roanoke | VA |
| SCD Assn | Bobbi Engram Foundation | Columbia | MD |
| SCD Assn | SCD Association of Maryland | Columbia | MD |
| SCD Assn | Greater Boston SCD Association | Dorchester | MA |
| SCD Assn | Sickle Cell Association of New Jersey | Newark | NJ |
| SCD Assn | Falling Angels Sickle Cell Foundation | Garnerville | NY |
| SCD Assn | Queens Sickle Cell Advocacy Network | Queens Village | NY |
| SCD Assn | Sickle Cell Thalassemia Patients Network | Brooklyn | NY |
| SCD Assn | Children's Sickle Cell Foundation | Pittsburgh | PA |
| SCD Assn | SCDAA-Philadelphia/Delaware Valley Chapter | Philadelphia | PA |
| SCD Assn | South Central PA Sickle Cell Council | Harrisburg | PA |
| SCD Assn | Sickle Association | Norfolk | VA |
| SCD Assn | [Sickle Cell Association of Richmond](http://sicklecell-richmond.org/) | Richmond | VA |
| Health Professional Assn | Maryland Academy of PAs | Millersville | MD |
| Health Professional Assn | Massachusetts Association of PAs | Westford | MA |
| Health Professional Assn | New Jersey State Society of Pas | Hackensack | NJ |
| Health Professional Assn | New York State Society of Pas | Menomonee Falls, WI | NY |
| Health Professional Assn | Pennsylvania Society of PAs | Greensburg | PA |
| Health Professional Assn | Virginia Academy of Pas | Charlottesville | VA |
| Health Professional Assn | American Assoc. of Nurse Practitioners |  | VT |
| Health Professional Assn | American Assoc. of Nurse Practitioners |  | MA |
| Health Professional Assn | American Assoc. of Nurse Practitioners |  | NY |
| Health Professional Assn | American Assoc. of Nurse Practitioners |  | North NY |
| Health Professional Assn | American Assoc. of Nurse Practitioners |  | South NY |
| Health Professional Assn | American Assoc. of Nurse Practitioners |  | NJ |
| Health Professional Assn | American Assoc. of Nurse Practitioners |  | PA |
| Health Professional Assn | American Assoc. of Nurse Practitioners |  | MD |
| Health Professional Assn | American Assoc. of Nurse Practitioners |  | PA |
| Health Professional Assn | American Assoc. of Nurse Practitioners |  | VA |
| Health Professional Assn | George Washington University NP program | Washington | DC |
| Health Professional Assn | Georgetown University NP program | Washington | DC |
| Health Professional Assn | Howard University NP program | Washington | DC |
| Health Professional Assn | The Catholic University Of America NP program | Washington | DC |
| Health Professional Assn | Coppin State University NP program | Baltimore | MD |
| Health Professional Assn | Salisbury University NP program | Salisbury | MD |
| Health Professional Assn | Jefferson College Of Health Sciences NP program | Roanoke | VA |
| AHEC | Eastern Virginia AHEC | Norfolk | VA |
| AHEC | Capital AHEC | Richmond | VA |
| AHEC | Virginia Statewide AHEC Program (VA AHEC) | Henrico | VA |
| AHEC | SE PA AHEC | Philadelphia | PA |
| AHEC | Southwest PA AHEC | Pittsburgh | PA |
| AHEC | Camden AHEC, Inc. | Camden | NJ |
| AHEC | Spanish Community Center/Shore AHEC | Landisville | NJ |
| AHEC | Brooklyn Queens Long Island AHEC | Brooklyn | NY |
| AHEC | Manhattan-Staten Island AHEC | New York | NY |
| AHEC | New York State AHEC System | Buffalo | NY |
| AHEC | Baltimore Area Health Education Center | Baltimore | MD |
| AHEC | Maryland AHEC Program | Baltimore | MD |
| Faith-based Org | Black Ministerial Alliance | Boston | MA |
| Faith-based Org | Interfaith Ministerial Alliance | Providence | RI |
| Health Professional Assn | MD Academy of Family Physicians |  | MD |
| Health Professional Assn | MA Academy of Family Physicians |  | MA |
| Health Professional Assn | NJ Academy of Family Physicians | Trenton | NJ |
| Health Professional Assn | NY Academy of Family Physicians | Albany | NY |
| Health Professional Assn | PA Academy of Family Physicians | Harrisburg | PA |
| Health Professional Assn | MD American College of Physicians | Baltimore | MD |
| Health Professional Assn | MA American College of Physicians |  | MA |
| Health Professional Assn | NJ American College of Physicians | Trenton | NJ |
| Health Professional Assn | NY American College of Physicians | Albany | NY |
| Health Professional Assn | PA American College of Physicians | Harrisburg | PA |
| Health Professional Assn | VA American College of Physicians |  | VA |
| Health Professional Assn | VA Academy of Family Physicians | Richmond | VA |
| Health Professional Assn | Black Nurses Rock - Delaware | Townsend | DE |
| Health Professional Assn | Black Nurses Rock - District of Columbia | DMV | DC |
| Health Professional Assn | Black Nurses Rock - Massachusetts | Boston | MA |
| Health Professional Assn | Black Nurses Rock - New Jersey |  | NJ |
| Health Professional Assn | Black Nurses Rock - Buffalo | Buffalo | NY |
| Health Professional Assn | Black Nurses Rock - NYC | New York City | NY |
| Health Professional Assn | Black Nurses Rock - Syracuse | Syracuse |  |
| Health Professional Assn | New England Regional BNA | Roxbury | MA |
| Health Professional Assn | Western Massachusetts Black Nurses Association | Springfield | MA |
| Health Professional Assn | Black Nurses Association of Greater Washington, DC Area | Washington | DC |
| Health Professional Assn | Downtown Baltimore Black Nurses Association | Baltimore | MD |
| Health Professional Assn | Black Nurses Association of Baltimore | Baltimore | MD |
